# A digital health peri-operative cognitive-behavioral intervention to prevent transition from acute to chronic postsurgical pain in adolescents undergoing spinal fusion (SurgeryPal^TM^): study protocol for a multisite randomized controlled trial

**DOI:** 10.1186/s13063-021-05421-3

**Published:** 2021-07-30

**Authors:** Jennifer A. Rabbitts, Chuan Zhou, Rocio de la Vega, Homer Aalfs, Caitlin B. Murray, Tonya M. Palermo

**Affiliations:** 1 Center for Clinical and Translational Research (CCTR), Seattle Children’s Hospital, 4800 Sand Point Way NE MB.11.500.3, Seattle, WA 98105 USA; 2 Department of Anesthesiology and Pain Medicine, University of Washington, 1959 NE Pacific St, Seattle, WA 98195 USA; 3 Center for Child Health Behavior and Development (CHBD), Seattle Children’s Hospital, 1920 Terry Avenue, Seattle, WA USA; 4 Department of Pediatrics, University of Washington, 4800 Sand Point Way NE, Seattle, WA 98105 USA; 5 Department of Psychology, University of Málaga, Campus de Teatinos, s/n, 29071 Málaga, Spain

**Keywords:** Adolescent scoliosis, Acute pain, Chronic pain, Psychosocial intervention, mHealth, Randomized controlled trial

## Abstract

**Background:**

Spinal fusion surgery is associated with severe acute postsurgical pain and high rates of chronic postsurgical pain in adolescents. Psychological distress, sleep disturbance, and low pain self-efficacy predict higher acute pain and likelihood of developing chronic postsurgical pain. Interventions targeting baseline psychosocial risk factors have potential to interrupt a negative trajectory of continued pain and poor health-related quality of life (HRQL) over time but have not yet been developed and evaluated. This randomized controlled trial will test effectiveness of a digital peri-operative cognitive-behavioral intervention (SurgeryPal^TM^) vs. education-control delivered to adolescents and their parents to improve acute and chronic pain and health outcomes in adolescents undergoing spine surgery.

**Methods:**

Adolescents 12–18 years of age undergoing spinal fusion for idiopathic conditions, and their parent, will be recruited from pediatric centers across the USA, for a target complete sample of 400 dyads. Adolescents will be randomized into 4 study arms using a factorial design to SurgeryPal^TM^ or education control during 2 phases of treatment: (1) pre-operative phase (one-month before surgery) and (2) post-operative phase (1 month after surgery). Acute pain severity and interference (primary acute outcomes) and opioid use will be assessed daily for 14 days following hospital discharge. Chronic pain severity and interference (primary acute outcomes), as well as HRQL, parent and adolescent distress, sleep quality, and opioid use/misuse (secondary outcomes), will be assessed at 3 months and 6 months post-surgery.

**Discussion:**

Demonstration of effectiveness and understanding optimal timing of perioperative intervention will enable implementation of this scalable psychosocial intervention into perioperative care. Ultimately, the goal is to improve pain outcomes and reduce reliance on opioids in adolescents after spine surgery.

**Trial registration:**

NCT04637802 ClinicalTrials.gov. Registered on November 20, 2020

**Supplementary Information:**

The online version contains supplementary material available at 10.1186/s13063-021-05421-3.

## Introduction

### Background and rationale

Spinal fusion surgery is one of the most frequently performed major musculoskeletal surgeries in children and adolescents, with an estimated 12,600 procedures performed in the USA annually [1]. The majority of pediatric spinal fusion surgeries are performed for idiopathic spinal deformities (e.g., scoliosis, spondylolisthesis), with adolescent idiopathic scoliosis being the most common. Spinal fusion results in intense acute postsurgical pain and high rates of chronic pain, with 80% of otherwise healthy adolescents reporting moderate to severe acute pain [2–4], and up to half reporting persistent pain 6–12 months post-surgery [5–7].

Recent biopsychosocial models conceptualize transition from acute to chronic pain as a complex interplay of biological, psychosocial, and sensory mechanisms [8, 9] and highlight the pre- and post-surgery phases as potential opportunities to intervene to prevent chronification of pain [10]. However, there is a lack of understanding of how to halt the transition from acute to chronic postsurgical pain (CPSP). In adolescents, psychosocial risk factors including anxiety and depressive symptoms, sleep disturbance, low pain self-efficacy, and high parent anxiety are associated with poorer pain and health outcomes at both short- and long-term follow-up after surgery [6, 11–15]. Moreover, adolescents who experience severe acute postsurgical pain are at elevated risk for developing CPSP, a condition marked by chronic pain which impacts health-related quality of life (HRQL) after surgery [14]. Interventions targeting baseline psychosocial risk factors may reduce acute postoperative pain and have potential to interrupt a negative trajectory of continued pain and poor health outcomes.

Limited research has evaluated peri-operative psychosocial interventions in adolescents undergoing spinal fusion [16]. The few existing pilot studies conducted to date have tested brief peri-operative interventions (e.g., relaxation strategies) demonstrating promising reductions in acute anxiety and pain intensity in the hospital [17] and in the first month following surgery [18]. However, there is a gap in measurement of longer-term outcomes to understand whether psychosocial interventions can also reduce occurrence of CPSP. Beyond the context of surgery, a large body of evidence demonstrates that cognitive-behavioral therapy (CBT) interventions are effective for reducing pain and disability in adolescents with existing chronic pain conditions [19]. CBT delivered before or after surgery may also be effective in *preventing* the transition from acute to CPSP.

Because of the numerous identified barriers (e.g., access, cost) to receiving psychosocial interventions in-person, digital health interventions have emerged as an effective solution to increase access to evidence-based interventions for pain [20–22], leading to improvements in pain and disability in adult and pediatric populations [23–26]. We hypothesize that this approach will also be effective for youth undergoing pediatric spinal fusion and will facilitate widespread dissemination and implementation.

### Objectives

This multicenter randomized controlled trial (RCT) aims to test the effectiveness of a digital CBT intervention called “SurgeryPal” in a factorial design comparing SurgeryPal^TM^ delivered to adolescents and their parents during one or both peri-operative phases (pre- vs. post-surgery) relative to online education (to control for participants’ time and attention). As recommended by PedIMMPACT, primary outcomes are acute and chronic pain severity and interference [27]. Secondary outcomes include HRQL, psychosocial distress, sleep disturbance, and opioid use/misuse. An additional aim will explore mechanisms of intervention effect on preventing CPSP, including reductions in acute pain severity and psychosocial distress.

## Methods

### Participants and setting

This is a phase II multicenter clinical trial and was approved by the Seattle Children’s Hospital (SCH) Institutional Review Board and registered at Clinicaltrials.gov (NCT04637802; see online supplementary file 1). This trial forms part of the National Institutes of Health (NIH) Helping to End Addiction Long-Term (HEAL) initiative, within the Pain Effectiveness Research Network (HEAL Pain ERN) [28], for which trial coordination is provided by the Recruitment and Trial Innovation Centers in the Trial Innovation Network.

The study sample will include adolescents ages 12–18 scheduled to undergo spinal fusion surgery for juvenile/adolescent idiopathic scoliosis, spondylolisthesis or kyphosis and their parents (or caregivers), with a target complete sample of 400 dyads. Complete inclusion and exclusion criteria are presented in Table 1. Adolescents and their parents will be referred to the study from surgical specialty hospitals across the USA that regularly conduct pediatric spinal fusion surgeries (see online supplementary file 2). Currently, there are 19 activated referring sites. Sites will continue to be added and onboarded, to reach recruitment goals, and we anticipate ~ 25 total sites.

**Table 1:** Eligibility criteria

**Inclusion criteria**	
Adolescent 12–18 years old at the time of enrollment.	
Adolescent undergoing scheduled spinal fusion surgery for one of the following eligible surgery indications: adolescent idiopathic scoliosis, juvenile idiopathic scoliosis, spondylolisthesis, or kyphosis.	
Parent or caregiver is the legal guardian and resides with the adolescent.	
**Exclusion criteria**	
Adolescent or parent does not speak English.	
Adolescent has severe learning disability, cognitive impairment or intellectual delay (i.e., is unable to read at 5th grade level or higher).	
Adolescent or parent does not have access to a smart device such as a smartphone, iPad, or tablet.	
Adolescent has a severe systemic disease (e.g., neuromuscular scoliosis, cancer).	
Adolescent takes medication daily for treatment of a chronic health condition, except for the following conditions: allergies, asthma, anxiety, and depression.	
Adolescent has a recent psychiatric admission within the past 30 days.	
Adolescent has a history of prior major surgery (open surgery, e.g., heart, lung, brain, or abdominal surgery, or prior spine surgery).	
Adolescent has a diagnosed chronic musculoskeletal pain condition (e.g., complex regional pain syndrome, fibromyalgia or widespread musculoskeletal pain).	

### Recruitment and enrollment

Providers at participating hospitals will approach adolescents and their parents at least 4 weeks prior to surgery to introduce the study. Providers will refer interested families directly to SCH study staff by securely sending families’ contact information, with permission, via the Research Electronic Data Capture system (REDCap) [29]. Families may also self-refer via the REDCap link, or by phone/email. SCH study staff will contact interested families to complete eligibility screening and enrollment via phone. Eligible families will provide consent (parents and adolescents age 18 years) and assent (adolescents < 18 years) verbally and using online forms in REDCap, including consent for sharing of deidentified data for use in future research (see online supplementary file 3).

### Trial design and randomization

**Fig. 1:**
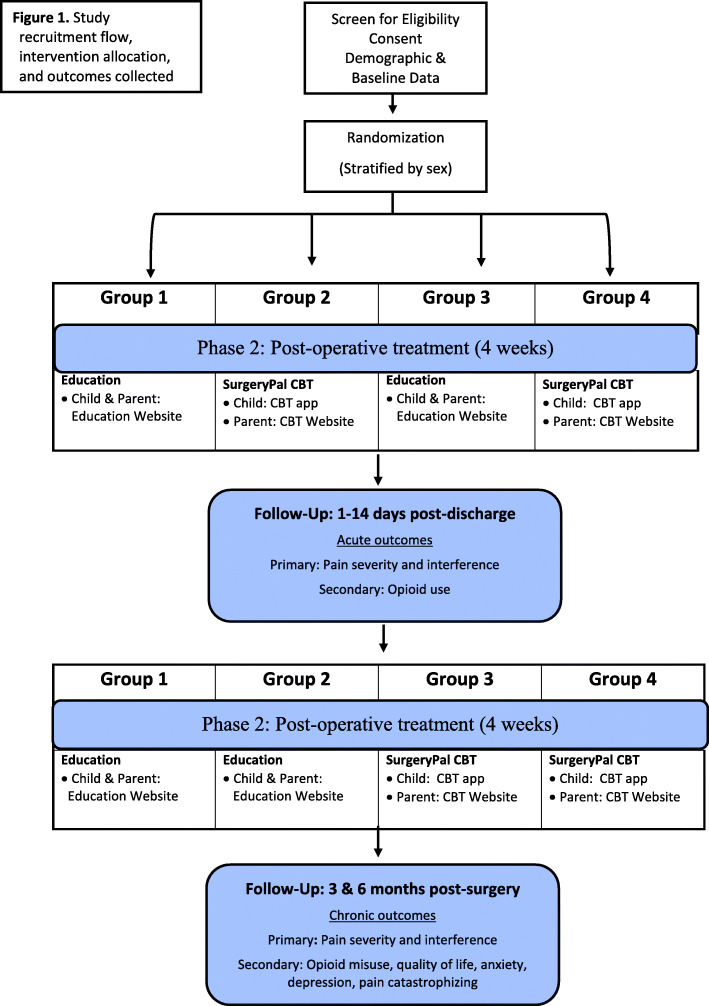
Study recruitment flow, intervention allocation, and outcomes collected

This study will utilize a single-blinded, randomized 2 × 2 factorial design (see Fig. 1). Block randomization will be used to allocate participants to one of four arms with random block sizes, stratified by participant sex. The randomization allocation table will be implemented using the REDCap randomization framework using an allocation ratio of 1:1:1:1 and will occur directly before treatment assignment. The randomization process will be overseen by an independent statistician, who is not involved with study procedures, including participant recruitment, intervention assignment, or any other forms of participant contact. Randomization results will only be directed to the study coordinator, who will assign participants to the intervention groups. All other study staff including the trial investigators, and data analyst will be blinded to treatment assignment. Care providers will not be informed of treatment assignment, and participation in the study will not affect clinical care. Participants may therefore engage in any other intervention or treatment (e.g., counseling, physical therapy). In the unlikely event of an adverse event, unmasking would be permissible.

We anticipate enrolling 500 adolescents to reach our target complete sample of 400 and thus expect 125 adolescents in each of the 4 study groups. Group 1 will receive education control during both pre- and post-operative phases and will not receive access to the SurgeryPal^TM^ CBT intervention. Group 2 will receive the pre-operative phase of the SurgeryPal^TM^ CBT intervention and post-operative education. Group 3 will receive pre-operative education and the post-operative phase of the SurgeryPal^TM^ CBT intervention. Group 4 will receive both pre- and post-operative phases of the SurgeryPal^TM^ CBT intervention. Parents will receive the SurgeryPal^TM^ CBT parent intervention or education control in the pre- and post-operative phases, corresponding to the adolescent’s group assignment.

### Interventions

Each pre- and post-surgery treatment phase has a duration of 4 weeks, with the pre-operative treatment phase beginning 4–6 weeks before surgery and the post-operative treatment phase beginning 3 weeks post-surgery. All components will be delivered via a website and a mobile app and are self-guided.

### Adolescent CBT intervention: SurgeryPal^TM^ mobile application

**Fig. 2:**
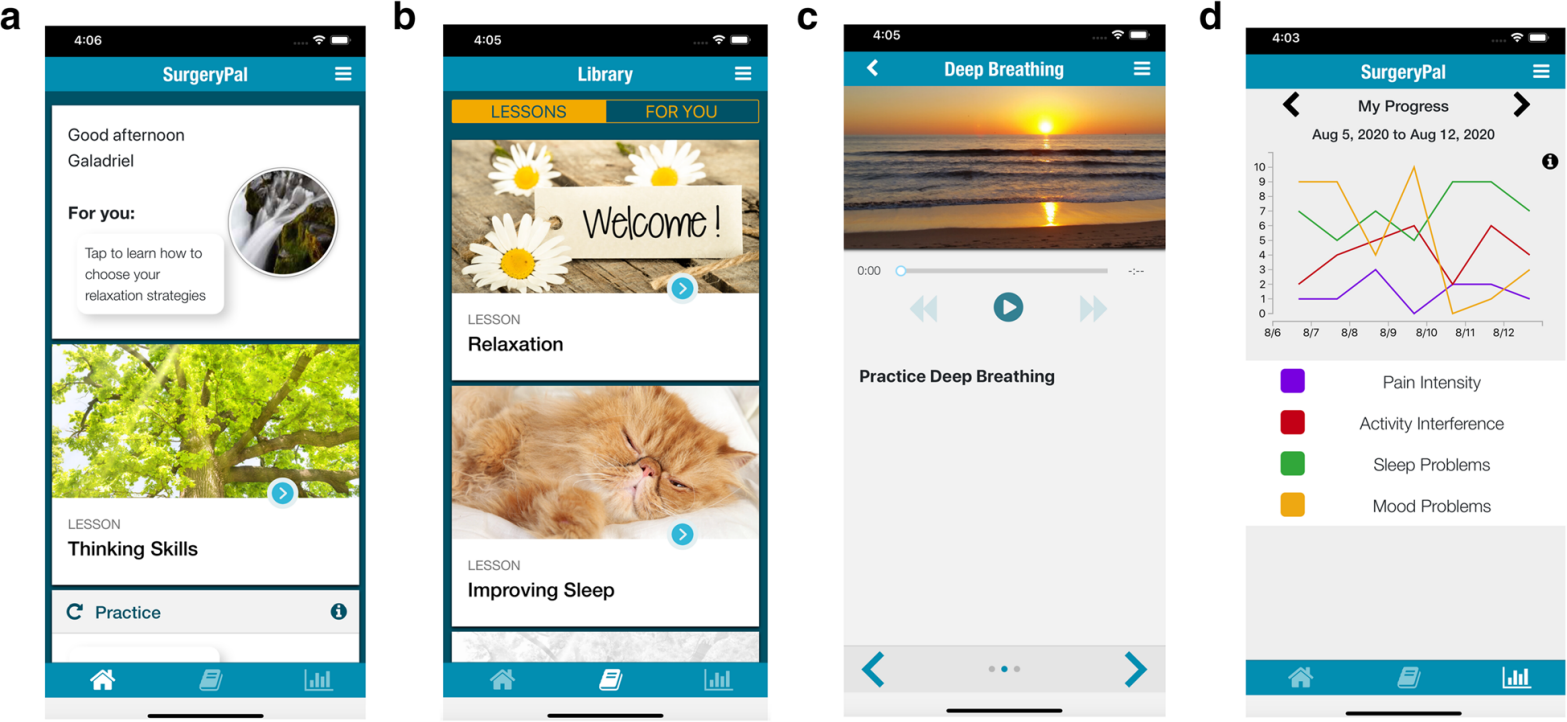
Screenshots from SurgeryPal teen mobile application showing **a** home screen, **b** lesson tab, **c** skills practice, and **d** symptom tracking

During the intervention phases, adolescents assigned to CBT intervention will be provided with access to the SurgeryPal^TM^ CBT program delivered via a mobile health application. Adolescents will download the app directly from iPhone or GooglePlay stores to their personal device and log in using a password. The intervention includes cognitive-behavioral strategies and resilience enhancing techniques that are intended to modify anxiety/distress, improve sleep, and reduce pain (Table 2). The mobile application has three functional components: (1) lessons, (2) cognitive behavioral “Skills Practice,” and (3) symptom tracking which triggers “For You” content (see Fig. 2).

Lessons are released weekly during each treatment phase and the intervention is intended to be completed in 3–4 weeks. The app delivers interactive instruction in cognitive skills (e.g., replacing negative thoughts, letting go of worries), relaxation strategies (e.g., deep breathing, imagery, progressive muscle relaxation), sleep hygiene (optimizing sleep duration and sleep quality), and pain coping skills (e.g., using relaxation and distraction to reduce pain). Each lesson’s theme is relevant to the timing of the perioperative period (e.g., “Getting ready for the hospital,” or “Coping at home after surgery”). Videos of adolescents describing their experiences and use of skills before or during recovery from spine surgery are incorporated throughout. Skills are available as guided audio recordings (e.g., mindful breathing) and interactive exercises (e.g., replacing negative thoughts). Adolescents rate emotions before and after practicing the skill and receive automated, tailored feedback based on whether emotions improve, worsen, or stay the same. The symptom tracking component allows adolescents to rate their anxiety/mood, sleep quality, activity limitations, and pain, each day using 11-point numerical rating scales which feeds into a customized graph that allows teens to track their symptoms and progress over time. Tailored “For You” material is released based on symptom ratings (e.g., “Routines for Better Sleep: Your Wind Down and Wake-Up Rituals” if participant reports poor sleep quality).

### Parent CBT intervention: SurgeryPal^TM^ website

During the intervention phase, the participating parent will receive access to the parent CBT website while their adolescent uses the SurgeryPal mobile application. The parent intervention provides instruction in cognitive-behavioral strategies to reduce parent anxiety/distress, as well as resilience-enhancing techniques for managing parent challenges with surgery (Table 2). Similar to the teen program, the parent website has 3 functional components: (1) lessons, (2) cognitive-behavioral “Skills Practice,” and (3) assignment review.

Parent lessons are released weekly and deliver interactive instruction in cognitive strategies (e.g., replacing negative thoughts) and relaxation strategies (e.g., deep breathing), as well as content on parent preparation before surgery (e.g., gathering information, talking to your teen before surgery) and strategies for recovery following surgery (e.g., importance of parent self-care). Lesson themes correspond to surgery timing (e.g., “Preparing for surgery” and “Coping at home after surgery”) and contain parent peer videos where parents discuss their experience and helpful strategies for coping with their adolescent’s spine surgery. Each lesson contains a parent assignment to practice and implement skills and strategies learned and triggers release of guided audio recording of skills to the practice section. Parents return to the website to complete interactive assignment review reporting on practice of skills/strategies, and receive tailored feedback based on their responses.

**Table 2:** Summary of adolescent and parent CBT intervention timing and content

Module	Adolescent content	Parent content
**Preoperative treatment phase (4 weeks duration, delivered 4–6 weeks prior to surgery)**		
**1**	**Preparing for surgery** -Introduction to relaxation strategies and training in deep breathing. -Information about the importance of good sleep for recovery from surgery and strategies to improve sleep habits (e.g., wind-down routine).	**Preparing for surgery** -Information about preparing for surgery and how to effectively gather information from the medical team. -Introduction to relaxation strategies and training in deep breathing. -Information about the importance of good sleep for recovery from surgery and strategies to support teen’s healthy sleep.
**2**	**Coping with worry before surgery** -Introduction to cognitive strategies. -Training in thought replacement and mindfulness.	**Coping with worry before surgery** -Introduction to cognitive strategies. -Training in thought replacement and mindfulness. -Strategies to enhance parent-teen communication.
**3**	**Getting ready for the hospital and recovery** -Information about preparing for the hospital and setting positive expectations. -Training in using imagery and distraction for managing anxiety or pain.	**Getting ready for the hospital and recovery** -Information about preparing for the hospital and setting positive expectations and social connections. -Training in using imagery for managing anxiety.
**Postoperative treatment phase (4 weeks duration, delivered 3–7 weeks post-surgery)**		
**1**	**Coping at home** -Information about the importance of adequate sleep and strategies to improve sleep habits (e.g., sleep schedule). -Information about the 4 P’s of pain management. -Training in music distraction and mindful breathing.	**Recovery at home after surgery** -Information about how to support healthy teen sleep habits and pain management. -Introduction to basic principles of self-care. -Training in mindful breathing.
**2**	**Getting back to activities** -Introduction to behavioral activation. -Training in strategies to gradually increase physical activities (e.g., activity pacing).	**Getting back to normal life after surgery** -Information about handling daily stress. -Information about how to support teen’s return to activities and school. -Training in progressive muscle relaxation to reduce distress.
**3**	**Long-term recovery** -Information about possible future challenges with recovery and education about chronic pain. -Training in progressive muscle relaxation.	**Long-term recovery** -Information about possible future challenges with recovery and education about chronic pain. -Training in using positive self-statements to reduce distress.

### Control condition: adolescent and parent education website

Participants assigned to the control condition will receive access to a patient education website developed for this study. The purpose of this condition is to control for time, attention, and online usage and to allow masking to treatment allocation. The education program provides youth and parents with basic information about pediatric spine surgery and what to expect during the acute and recovery phases. Content was compiled from publicly available educational websites about spinal fusion surgery (e.g., Kids Health, Boston Children’s Hospital, Scoliosis Research Society, MyHealth Alberta, WebMD), and does not include any instruction in the behavioral and cognitive skills taught within the SurgeryPal^TM^ program. In our prior RCTs using similar education control sites, parents and youth have shown a high level of engagement and high ratings of treatment credibility [24].

### Criteria for discontinuing allocated interventions

Adverse events, if any, are expected to be minor and there are no planned criteria for discontinuing or modifying allocated interventions. Participants will be removed from the study by the trial investigators if they are non-compliant with the pre-randomization baseline assessment, undergo a second major surgery (e.g., repeat spinal fusion surgery) or are diagnosed with a new complex medical condition (e.g., cancer) during the study time frame. Timepoints prior to the new presentation of exclusionary condition will be analyzed. Participants may withdraw from the study and intervention at any time.

### Treatment fidelity and strategies to improve adherence to interventions

Treatment is standardized for all participants because the intervention is delivered via a web program and mobile application. Research staff will interact with participants regularly to communicate study procedures, encourage program completion, and address general questions/technical issues. Automated reminders using push notifications in the SurgeryPal^TM^ mobile application will also promote adherence to the intervention. Analytics will be used to track participants’ logins, lesson completion, and assignment completion in the web program as well as download and usage of the SurgeryPal^TM^ mobile application.

### Assessment plan

All assessments will be completed online through the REDCap database [29] via email/text survey links. Youth and parents will complete a baseline assessment prior to randomization (T1, baseline). Following hospital discharge from surgery, adolescents will complete daily assessments for 14 days (T2, acute outcomes). Adolescents will report on daily pain outcomes for 7 days and adolescents and parents will complete additional outcome measures at 3 months (T3, chronic outcomes) and 6 months (T4, final follow-up) post-surgery. Participants will receive email or phone reminders by study staff to complete survey measures. Automated REDCap alerts will also be used to inform study coordinators about missing data so they can follow-up with participants to obtain responses. Additionally, participants are provided with an Amazon gift card for completing each assessment.

### Primary outcome measures

*Pain severity and interference.* Primary acute and chronic outcomes will be assessed using the 24-h (short) version of the Brief Pain Inventory (BPI), a validated patient-reported outcome measure comprised of a pain severity scale (4 items) and a pain interference scale (7 items) [30]. Adolescents will complete this measure daily for 14 days beginning on day 1 after hospital discharge (T2) and will complete the measure daily for 7 days at the follow-up assessments (T3 and T4). Adolescents rate worst, least, average, and current pain severity and rate pain interference on activity, mood, walking, work/school, relations, sleep, and enjoyment of life in the preceding 24 h. Responses are indicated on numerical rating scales with anchors 0 = “No Pain”/“Does not Interfere” and 10 = “Pain as bad as you can imagine”/“Completely Interferes”. The pain severity scale (average of 4 items) and pain interference scale (average of 7 items) will be calculated for each diary day completed. The BPI has been used to assess postsurgical pain intensity and interference in adolescents after major surgery [31].

The acute pain primary outcomes (*acute pain severity and interference)* will be derived as the individual rate of recovery based on BPI trajectories separately for pain severity and pain interference over 14 days immediately post-discharge (T2). The chronic pain primary outcomes (c*hronic pain severity and interference*) will be assessed as the mean of the participant’s daily pain ratings on the BPI separately for pain severity and pain interference at 3 months post-surgery (T3).

### Secondary outcome measures

Table 3 provides details on the secondary outcome measures.

**Table 3:** Secondary outcomes measures

Measure	Description	Respondent	Time point			
			T1	T2	T3	T4
Pediatric Quality of Life Inventory - Short form, acute version^a^ [32, 33]	15-item measure assessing health-related quality of life (HRQL) over the last 7 days across the following domains/subscales: physical health, psychosocial health, and total HRQL, reported on a 5-point Likert scale.	Adolescent	✔		✔	✔
Opioid use [30]	2 items on the BPI assessing daily opioid use and pain relief from medications. Acute opioid use will be calculated as the number of days of opioid use during 14 days daily monitoring (T2).	Adolescent	✔	✔	✔	✔
NIDA-Modified ASSIST Tool^a^ [34, 35]	15-item measure assesses alcohol, smoking, substance, and opioid misuse in the past 2 weeks; additional questions about reasons for misuse (e.g., to treat pain, to get high, etc.) and source of opioids (e.g., prescription, from a friend or relative) will be used from the National Survey on Drug Use and Health [36].	Adolescent	✔		✔	✔
PROMIS Pediatric Anxiety SF8a [37]	8-item measure used to assess adolescent self-reported anxiety. Adolescents endorse frequency of fear, anxiety, hyperarousal, and somatic symptoms experienced in the prior 7 days, on a 5-point Likert scale.	Adolescent	✔		✔	✔
PROMIS Pediatric Depression SF8a [37]	8-item measure assess depressive symptoms. Frequency of negative mood, negative views of self, decreased positive affect, and decreased engagement in the prior 7 days reported on a 5-point Likert scale.	Adolescent	✔		✔	✔
Patient Health Questionnaire-4^a^ [38]	4-item screening measure of general anxiety and depressive symptoms experienced in the preceding 2 weeks by the respondent.	Adolescent, parent	✔		✔	✔
Pain Catastrophizing Scale – Child Version^a^ [39]	13-item measure that assesses child pain catastrophizing. Adolescents report frequency of symptoms of rumination, magnification, and helplessness experienced when in pain using a 5-point Likert scale.	Adolescent	✔		✔	
Pain Catastrophizing Scale – Parent Version^a^ [40]	13-item measure that assesses parents’ symptoms of rumination, magnification, and helplessness, in response to their child experiencing pain, on a 5-point Likert scale.	Parent	✔		✔	
Adolescent Sleep Wake Scale 10^a^ [41]	10–item measure of self-reported sleep quality in preceding month, including going to bed, falling asleep, reinitiating sleep, and returning to wakefulness using a 6-point Likert scale; four additional items from the Children’s Report of Sleep Patterns (CRSP) measure querying bedtime and wake time on weekdays and weekends will assess sleep duration [42].	Adolescent	✔		✔	✔
Patient Global Impression of Severity Scale^a^	1-item assessing adolescent’s self-reported global impression of pain severity in the past 24 h (T2) or past week (T3 and T4), with response options “None” (0), “Mild” (1), “Moderate” (2), and “Severe” (3)	Adolescent		✔	✔	✔

^a^Pediatric core Common Data Element (CDE) measures for the Help End Addiction Long Term (HEAL) Initiative. PROMIS, Patient-Reported Outcome Measurement Information System; NIDA, National Institute on Drug Abuse; ASSIST, Alcohol, Smoking and Substance Involvement Screening Test

T1, pre-randomization baseline; T2, acute outcomes daily for 14 days post-hospital discharge; T3, chronic outcomes at 3 months post-surgery; T4, final follow-up at 6 months post-surgery

### Covariates

Parents will report on socio-demographic factors (age, sex, and race/ethnicity), and adolescents will report on pre-surgery pain severity and interference using the 7-day recall version of the Brief Pain Inventory [43].

### Treatment variables

*Treatment satisfaction and acceptability.* Adolescents and parents will complete the Treatment Evaluation Inventory Short Form, a 9-item self-report measure assessing perceptions of acceptability and satisfaction with the pre- and post-operative treatment content and approach [44], directly after each treatment phase. Select items on this measure were adapted to be specific to treatment of pediatric postsurgical pain. Items are rated on a 5-point scale (1 = strongly disagree, 5 = strongly agree) and summed to create a total score, with higher scores indicating higher levels of treatment satisfaction and acceptability.

*Treatment experience*. Adolescents and parents will complete a Treatment Experience Survey developed for this study concerning whether they experienced negative effects of study participation (e.g., worrying about pain, overwhelmed with treatment assignments, tension at home) and rate the level of discomfort experienced (0 = “did not affect me/my teen at all” to 3 = “affected me/my teen very negatively”). The number, type, and severity of negative experiences will be summarized. Participants will also report unrelated adverse events via free text response.

*Concomitant therapy.* Parents will report on additional health services accessed for pain or anxiety management during the pre- and post-operative treatment phases. Responses will be coded into a binary variable indicating whether concomitant therapy was accessed (yes/no).

### Data management

Research data will be stored on a Health Insurance Portability and Accountability Act (HIPAA)-compliant secure server hosted, managed, and monitored by the University of Utah Data Coordinating Center (DCC), with daily backups, and will be deidentified at the earliest possible opportunity. Participant contact information will be stored separately in secure, password-protected databases stored locally on HIPAA-compliant servers at Seattle Children's Research Institute and the University of Washington Institute of Translational Health Sciences. Databases will be linkable only by a unique study identifier.

### Data analysis

The primary analyses will be intent-to-treat analyses which will include all participants randomized, with adolescent as the unit of analysis. All regression analyses will adjust for a priori identified demographic variables (age, sex, race/ethnicity), prognostic covariates (pre-surgery pain intensity), and study site. Separate models will be conducted to analyze pain severity and pain interference (primary outcomes), as assessed by the BPI. For multi-item instruments, published rules on treatment of missing data will be followed where available; otherwise, hot deck imputation will be used for individual items for less than 20% missing data. There are no interim analyses planned.

### Primary efficacy outcomes

*Hypothesis 1 (acute outcomes): Adolescents receiving pre-operative CBT will have a faster rate of recovery of pain severity and interference during the 14 days following hospital discharge, compared to adolescents who receive pre-operative education.* The acute outcomes will be assessed before any post-operative intervention has taken place. Therefore, arms 1 and 3 will be combined into the control group (pre-operative education); arms 2 and 4 will be combined into the intervention group (receiving pre-operative CBT intervention) for this analysis (see Table 4). We will apply nonlinear mixed effects models with an exponential decay functional form, to evaluate individual rate of recovery in daily pain severity and interference, or how quickly the process decays/improves from the initial value to the asymptote. Rate parameters will be estimated using restricted maximum likelihood (REML) approach and compared using multivariate Wald test. As a further primary analysis, we will treat daily pain severity and interference as repeated measures using generalized estimating equations (GEE) to account for clustering within participants.

**Table 4:** Treatment arms

Group	Pre-operative phase	Post-operative phase
1	ED	ED
2	CBT	ED
3	ED	CBT
4	CBT	CBT

*ED* online education, *CBT* Cognitive Behavioral Therapy Intervention

*Hypothesis 2 (chronic outcomes): Adolescents receiving post-operative CBT alone will have lower pain severity and interference, compared to adolescents receiving education control at 3 months.* We will consider the education control arm (arm 1) as the control group and post-operative CBT alone arm (arm 3) as the intervention group. Covariates adjusted linear regression models will examine group differences in average pain severity and interference, as continuous outcomes, at 3 month follow-up.

*Hypothesis 3 (chronic outcomes): Adolescents receiving both phases of peri-operative CBT will have lower pain severity and interference compared to adolescents receiving single phase (pre- or post-surgery) intervention or education control alone at 3 months.* We will apply linear regression models to examine differences in average pain severity and interference between individual study arms, treated as a 4-level categorical predictor variable.

### Secondary analyses

*Secondary efficacy outcomes:* Additional covariates adjusted regression models will examine differences in secondary acute outcomes (opioid use; T2), and secondary chronic outcomes (HRQL, anxiety, depressive symptoms, sleep quality, opioid misuse; T3) between the relevant treatment arms (as noted in primary analyses).

*Exploratory analyses:* Additional logistic analyses will compare rates of CPSP, a binary variable based on pain intensity and HRQL, at 3 months and 6 months in youth receiving combined pre- and post-operative CBT (arm 4) compared to arms 1, 2, and 3. We will also explore extent through which acute post-operative pain intensity and interference (T2) as well as changes in psychosocial factors (sleep quality, anxiety, and depression; T2-T4 relative to T1) exert their influence on (i.e., mediate) change in pain intensity, HRQL, and pain interference at 3 months follow-up using a structural equation modeling (SEM) approach.

Effect modification will be examined through regression models with interactions and subgroup analysis testing whether there are differential intervention effects across sex, racial, and ethnic groups on primary and secondary acute and chronic outcomes.

### Sample size and power calculations

Power calculations on primary pain outcomes are based on data available from our preliminary peri-operative studies [4, 7, 31] and from prior peri-operative intervention studies [16–18]. Power analyses for primary acute outcomes were based on rate of recovery using an exponential decay model. Based on pilot data, we assume log(rate) = − 1.21 (SE = 0.38) in the intervention arm, which translates into a half-life (days taken to achieve 50% reduction in BPI outcome from time 0) of 4.13 days. Using a conservative estimate of 35% increased half-life of recovery in the control arm, 31 subjects per arm would provide 90% power; N = 24/arm for 80% power.

For the second acute parameter of interest, power calculations were performed using GEE model on time-averaged difference (TAD) using individual daily repeated measures of BPI scores. In order to detect a group difference of 1.2 with standard deviation 2.0, we need to enroll 199 participants into each of the two comparison groups (total sample of 398; assuming 1:1 allocation of subjects into pre-operative education vs. intervention, within-subject observations follow AR(1) correlation pattern with a correlation coefficient of 0.7, and a missing completely at random rate of 5% each day).

For the primary chronic pain outcomes, to detect a group difference of 1.0 with standard deviation 1.8, 35 subjects are needed per arm for 80% power based on two-sample t test.

The target complete sample is 400 adolescent/parent dyads. Conservatively accounting for 20% attrition, we plan to enroll 500 adolescents but will adjust this if completion rates are > 80%.

### Oversight and monitoring

Study monitoring to ensure proper consenting and assenting procedures, maintenance of IRB approval, intervention implementation, and security of databases will be conducted by Trial Innovation Centers (TICs) including the University of Utah DCC and Duke/Vanderbilt Clinical Coordinating Centers (CCCs). The DCC will work closely with research coordinators and principal investigators to oversee study data management. In addition, an independent Data and Safety Monitoring Board (DSMB) comprised of experts from pediatric psychology, behavioral interventions, and statistics will convene biannually in an open session followed by a closed session to review study progress, data, and participant safety. Authorship will be determined in accordance with the HEAL Initiative Publication Policy.

## Discussion

This study will address a critical gap in knowledge of perioperative interventions targeting psychosocial risk factors to prevent transition from acute to CPSP in adolescents undergoing spinal fusion surgery. Our trial will use an innovative factorial design to test two intervention phases, pre-operative and post-operative, in order to determine the optimal timing of intervention delivery. This study will build on prior research examining short-term efficacy of peri-operative psychosocial interventions to reduce acute pain intensity by testing improvement on clinically meaningful and longer-term post-surgical outcomes such as chronic pain interference and quality of life. In addition, our recruitment design across multiple pediatric spine centers allows for broad inclusion of youth undergoing spinal surgery at community and academic hospital sites in many different regions of the USA.

To engage wide participation in a peri-operative program, interventions must use flexible, accessible, low-cost delivery models. Research with family stakeholders indicates that teens and parents prefer technology-based delivery within the current system of peri-operative care [45]. To address these needs, we developed the CBT and education interventions to be flexibly delivered via a mobile application and website for deployment in the trial. If successful, following the trial, we will have the unique opportunity for widespread dissemination of the SurgeryPal^TM^ interventions to adolescents receiving care in pediatric spine centers across the country.

The USA currently faces two national health crises relevant to perioperative pain management: rising rates of chronic pain, and an epidemic of opioid addiction and overdose, both of which extend to adolescents [46–49]. Neurobiological and psychosocial changes occurring during adolescence increase vulnerability to both drug addiction [50] and chronic pain [51]. Pain problems and opioid use/misuse often begin in adolescence and place individuals at risk for lifelong problems [52, 53]. Thus, prevention strategies addressing these health crises must address risk during this vulnerable developmental period. Major musculoskeletal surgeries, such as spinal fusion, are frequently timed for the adolescent period, and are associated with high rates of chronic postsurgical pain. Moreover, opioids are the cornerstone for pain management after these surgeries, and opioid-naïve youth undergoing surgery are at elevated risk for developing persistent opioid use over the subsequent year [54]. Adolescents undergoing major surgeries urgently need effective therapies to reduce pain, in order to mitigate potential for chronic pain, and to reduce exposure to opioids.

By studying a model of prevention during two intervention phases, this trial will take a critical step in pain research by examining whether psychosocial interventions implemented before the inciting event (primary prevention) and/or during the recovery phase (secondary prevention) may prevent acute to chronic pain transition. In addition, this RCT is designed to explore mechanisms underlying treatment effect, which will advance understanding of the transition from acute to chronic pain.

## Trial status

Recruitment started December 2020. The current approved protocol is version 1.08 (Initial submission approval date: 07 April 2020; approved version at final journal submission: 28 April 2021). Recruitment is estimated to be complete by December 2023. Any protocol modifications will be updated on ClinicalTrials.gov and on the participants’ consent form.

## Supplementary Information

**Additional file 1.** Recommended items to address in a clinical trial protocol and related documents.

**Supplementary file 1.** World Health Organization Trial Registration Data Set.

**Supplementary file 2.** Participating SurgeryPal Referral Sites (as of 6/30/2021*).

**Supplementary file 3.** Consent and Assent Forms.

## Data Availability

The study protocol has been reported in accordance with the Standard Protocol Items: Recommendations for Clinical Interventional Trials (SPIRIT) guidelines (Additional file 1). The data collection forms are available from the corresponding author on request. A database for underlying primary data for publications will be made publicly available on the NIH HEAL Initiative central data repository. The database will be de-identified in accordance with the definitions provided in the Health Insurance Portability and Accountability Act (HIPAA) and will be accompanied by a data dictionary that provides a concise definition of every data element included in the database. The policies for release of and access to this database are in accordance with the HEAL Data Sharing policy as determined by the NIH. Results of this trial will be published in international peer-reviewed journals by the study team.

## Abbreviations

BPI: Brief Pain Inventory
CPSP: Chronic postsurgical pain
