# Correction to: A digital health peri-operative cognitive-behavioral intervention to prevent transition from acute to chronic postsurgical pain in adolescents undergoing spinal fusion (SurgeryPal^TM^): study protocol for a multisite randomized controlled trial

**DOI:** 10.1186/s13063-021-05596-9

**Published:** 2021-09-21

**Authors:** Jennifer A. Rabbitts, Chuan Zhou, Rocio de la Vega, Homer Aalfs, Caitlin B. Murray, Tonya M. Palermo

**Affiliations:** 1 Center for Clinical and Translational Research (CCTR), Seattle Children’s Hospital, 4800 Sand Point Way NE MB.11.500.3, Seattle, WA 98105 USA; 2 Department of Anesthesiology and Pain Medicine, University of Washington, 1959 NE Pacific St, Seattle, WA 98195 USA; 3 Center for Child Health Behavior and Development (CHBD), Seattle Children’s Hospital, 1920 Terry Avenue, Seattle, WA USA; 4 Department of Pediatrics, University of Washington, 4800 Sand Point Way NE, Seattle, WA 98105 USA; 5 Department of Psychology, University of Málaga, Campus de Teatinos, s/n, 29071 Málaga, Spain

**Correction to: Trials 22, 506 (2021)**

**https://doi.org/10.1186/s13063-021-05421-3**

Following the publication of the original article [1], we were notified of an error in Fig. 1, the embedded SPIRIT diagram. In the first horizontal blue box, the text should read: “Phase 1: Pre-operative treatment (4 weeks)”.

The original paper has been corrected.
