# Supplementary material for: A digital health peri-operative cognitive-behavioral intervention to prevent transition from acute to chronic postsurgical pain in adolescents undergoing spinal fusion (SurgeryPalTM): study protocol for a multisite randomized controlled trial
Source: Trials. 2021 Jul 30;22:506. doi: 10.1186/s13063-021-05421-3 (PMC8325315; doi:10.1186/s13063-021-05421-3)
Supplement: Supplementary file 2 — Supplementary file 1. World Health Organization Trial Registration Data Set. [file 13063_2021_5421_MOESM2_ESM.docx]

**Supplementary Table 1. World Health Organization Trial Registration Data Set.**

| **Data category** | **Information** |
| --- | --- |
| Primary registry and trial identifying number | ClinicalTrials.gov NCT04637802 |
| Date of registration in primary registry | November 20, 2020 |
| Secondary identifying numbers | UG3 HD102038, UH3 HD102038 ([U.S. NIH Grant/Contract)](https://projectreporter.nih.gov/reporterapi.cfm?PROJECTNUM=UH3HD102038&Fy=all) |
| Source(s) of monetary or material support | National Institutes of Health |
| Primary sponsor | *Eunice Kennedy Shriver* National Institute of Child Health & Human Development and National Institute of Neurological Disorders and Stroke of the National Institutes of Health |
| Secondary sponsor | National Center for Advancing Translational Sciences of the National Institutes of Health (U24TR001597, U24TR001608, U24TR001609, and U24TR001579). |
| Contact for public queries | Jennifer A. Rabbitts, MD, 206-987-2704, jennifer.rabbitts@seattlechildrens.org |
| Contact for scientific queries | Jennifer A. Rabbitts, MD, 206-987-2704, jennifer.rabbitts@seattlechildrens.org |
| Public title | Digital Health Psychosocial Intervention for Adolescent Spine Surgery Preparation and Recovery (SurgeryPal) |
| Scientific title | Effectiveness of an mHealth psychosocial intervention to prevent transition from acute to chronic postsurgical pain in adolescents |
| Countries of recruitment | United States |
| Health condition(s) or problem(s) studied | Juvenile Scoliosis, Idiopathic Scoliosis; Adolescent Scoliosis; Congenital Scioliosis; Kyphosis; Spondylolisthesis |
| Intervention(s) | Experimental: SurgeryPal Cognitive Behavioral Therapy intervention  Active comparator: Education control |
| Key inclusion and exclusion criteria | Inclusion Criteria:  Teen  •12 to 18 years old at the time of enrollment  •Undergoing scheduled spinal fusion surgery for eligible condition  Parent/Caregiver  •Parent or legal guardian of child who meets study criteria  Exclusion Criteria:  Teen  •Does not speak or understand English  •Has severe learning disability, cognitive impairment or intellectual delay  •Recent psychiatric admission (in the past 30 days)  •Severe systemic disease  •Takes medication daily for treatment of a chronic medical condition (except allergies, asthma, anxiety, depression)  •Prior major surgery (open surgery, or prior spine surgery)  •Diagnosed chronic musculoskeletal pain condition (e.g. complex regional pain syndrome, fibromyalgia, widespread musculoskeletal pain)  Parent/Caregiver  •Does not speak or understand English |
| Study type | Interventional Allocation: randomized Intervention model: factorial assignment Masking: care provider, investigator, outcomes assessor Primary purpose: treatment Phase II |
| Date of first enrolment | December 2020 |
| Target sample size | 400 |
| Recruitment status | Recruiting |
| Primary outcome(s) | Post-surgical pain intensity and interference [Time Frame: Up to 3 weeks post-surgery]  Chronic pain intensity and interference [Time Frame: 3 months post-surgery] |
| Key secondary outcomes | Change in health-related quality of life [Time Frame: Baseline, 3-months post-surgery, 6-months post-surgery]  Change in opioid use [Time Frame: Baseline, Up to 3 weeks post-surgery, 3-months post-surgery, 6-months post-surgery]  Change in opioid misuse [Time Frame: Baseline, 3-months post-surgery, 6-months post-surgery]  Change in psychosocial distress [Time Frame: Baseline, 3-months post-surgery, 6-months post-surgery]  Change in mental health [Time Frame: Baseline, 3-months post-surgery, 6-months post-surgery]  Change in sleep quality [Time Frame: Baseline, 3-months post-surgery, 6-months post-surgery]  Change in pain catastrophizing [Time Frame: Baseline, 3-months post-surgery]  Global pain severity [Time Frame: Up to 3 weeks post-surgery, 3-months post-surgery, 6-months post-surgery] |
|  |  |
