# Supplementary material for: A digital health peri-operative cognitive-behavioral intervention to prevent transition from acute to chronic postsurgical pain in adolescents undergoing spinal fusion (SurgeryPalTM): study protocol for a multisite randomized controlled trial
Source: Trials. 2021 Jul 30;22:506. doi: 10.1186/s13063-021-05421-3 (PMC8325315; doi:10.1186/s13063-021-05421-3)
Supplement: Supplementary file 3 — Supplementary file 2. Participating SurgeryPal Referral Sites (as of 6/30/2021*). [file 13063_2021_5421_MOESM3_ESM.docx]

**Supplementary Table 1. Participating SurgeryPal Referral Sites (as of 6/30/2021*)**

| **Site** | **Location** |
| --- | --- |
| American Family Children’s Hospital | Madison, WI |
| Children’s Mercy Kansas City | Kansas City, MO |
| Connecticut Children’s Medical Center | Hartford, CT |
| Dayton Children’s Hospital | Dayton, OH |
| Duke Children's Hospital | Durham, NC |
| Hassenfeld Children’s Hospital | New York, NY |
| Johns Hopkins Children's Center | Baltimore, MD |
| Midwest Orthopaedics at Rush | Chicago, IL |
| Nemours A.I. Dupont Hospital for Children | Wilmington, DE |
| Nemours Children's Specialty Care, Jacksonville | Jacksonville, FL |
| OHSU Doernbecher Children's Hospital | Portland, OR |
| Seattle Children’s Hospital | Seattle, WA |
| Shriners Hospitals for Children - Chicago | Chicago, IL |
| Tufts Children's Hospital in Boston | Boston, MA |
| UNC Children's Hospital | Chapel Hill, NC |
| Arkansas Children's Hospital | Little Rock, AR |
| UCLA Mattel Children's Hospital | Los Angeles, CA |
| Children's Hospital of Richmond at VCU | Richmond, VA |
| Wake Forest Baptist Health: Brenner Children's | Wake Forest, NC |

*Referral sites continue to be added
