# Supplementary material for: A digital health peri-operative cognitive-behavioral intervention to prevent transition from acute to chronic postsurgical pain in adolescents undergoing spinal fusion (SurgeryPalTM): study protocol for a multisite randomized controlled trial
Source: Trials. 2021 Jul 30;22:506. doi: 10.1186/s13063-021-05421-3 (PMC8325315; doi:10.1186/s13063-021-05421-3)
Supplement: Supplementary file 4 — Supplementary file 3. Consent and Assent Forms. [file 13063_2021_5421_MOESM4_ESM.pdf]

Assent Form

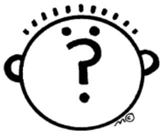

What is a research study?

Research studies help us learn new things. We can test new ideas. First, we ask a question. Then we try to find the answer.

This paper talks about our research and the choice that you have to take part in it. We want you to ask us any questions that you have. You can ask questions any time.

Important things to know…

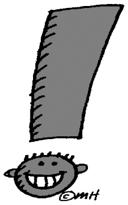

- You get to decide if you want to take part.
- You can say 'No' or you can say 'Yes'.
- No one will be upset if you say 'No'.
- If you say 'Yes', you can always say 'No' later.
- You can say 'No' at anytime.
- We would still take good care of you no matter what you decide.

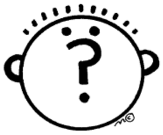

Why are we doing this research?

We are doing this research to find out more about how to help kids feel better before and after they have surgery.

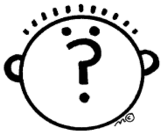

What would happen if I join this research?

If you decide to be in the research, we would ask you to do the following:

- Questions: We would ask you to read some questions. Then you would give your answers.
- Talking: A person on the research team would ask you some questions. Then you would say your answers out loud.
- Website/App: We would ask you to use a website or phone app regularly before and after your surgery.

Seattle Children's IRB Approved
Effective: 2/18/2021

Could bad things happen if I join this research?

Some of the questions might make you uncomfortable or be hard to answer. We will try to make sure that no bad things happen. You can skip questions you do not want to answer.

You can say ‘no’ to what we ask you to do for the research at any time and we will stop.

Could the research help me?

We think being in this research may help you because it might prepare you for your surgery, and help you feel better after surgery. And someday we hope it will help other kids who have surgery like you.

What else should I know about this research?

If you don’t want to be in the study, you don’t have to be.

It is also OK to say yes and change your mind later. You can stop being in the research at any time. If you want to stop, please tell the research doctors.

To thank you for being in the study, we would give you up to $340. You should talk with your parents about how you would like to use this.

You can ask questions any time. You can talk to Dr. Rabbitts, Dr. Palermo, or one of the research coordinators. Ask us any questions you have. Take the time you need to make your choice.

Is there anything else?

If you want to be in the research after we talk, please type your name below. This shows we talked about the research and that you want to take part.

Seattle Children's IRB Approved
Effective: 2/18/2021

PARENTAL PERMISSION FORM
CONSENT FORM: Ages 18 and up
ASSENT FORM: Ages 13-17

Study Title: SurgeryPal - Effectiveness of the SurgeryPal intervention in adolescents undergoing major surgery

Principal Researchers: Jennifer Rabbitts, MD and Tonya Palermo, PhD

The Research Team:

Name/Degree	Phone Number	E-mail
Jennifer Rabbitts, MD	206-884-1361	Jennifer.rabbitts@seattlechildrens.org
Tonya Palermo, PhD	206-884-4208	Tonya.palermo@seattlechildrens.org
Homer Aalfs, BS	206-884-1845	Homer.aalfs@seattlechildrens.org
Shannon Higgins, BS	206-884-0580	Shannon.higgins@seattlechildrens.org

If you have questions about your rights as a research study participant, you can call the Institutional Review Board at (206) 987-7804.

Key Information: You have the option to take part in a research study. This is a consent, assent, and parental permission form. The goal of this form is to give potential participants the information they need to decide whether to participate in the research. The first portion of this form includes a summary of the key information about the research study. Participation in the study is voluntary.

Potential Participants 18 years and older: This is a consent form. It provides a summary of the information the research team will discuss with you. If you decide that you would like to take part in this research study, you would sign this form to confirm your decision. If you sign this form, you will receive a signed copy of this form for your records.

Potential Teen Participants: This form serves as an assent form. That means that if you choose to take part in this research study, you would sign this form to confirm your choice. Your parent or legally authorized representative would also need to give their permission and sign this form for you to join the study.

Parents/Legally authorized representatives: You have the option of having your teen join a research study. This is a parental permission form. It provides a summary of the information the research team will discuss with you. If you decide that your teen can take part in this study, you

Seattle Children's IRB Approved
Effective: 4/28/2021

would sign this form to confirm your decision. If you sign this form, you will receive a signed copy for your records.

Joining the study as a parent (and/or Legally Authorized Representative): Parents have the option to take part in this research study. This form also serves as a consent form for parent participation. The word "you" in this form may refer to you and/or your child.

If you are interested in participating after reviewing the key information below, continue through the portion of the form containing the "Detailed Information" about the study. Feel free to take notes, write questions or highlight any part of this form

What should I know about this study?

- This form explains what would happen if you join this research study.
- Please read it carefully. Take as much time as you need.
- Please ask the research team questions about anything that is not clear.
- You can ask questions about the study any time.
- If you choose not to be in the study, it will not affect your medical care at your hospital.
- If you say 'Yes' now, you can still change your mind later.
- You can quit the study at any time.
- You would not lose benefits or be penalized if you decide not to take part in the study or to quit the study later.
- This study is being supported by funding from the National Institutes of Health (NIH).

What is the purpose of this study?

The goal of any research study is to answer questions. We (the research team listed on the front of this form and our staff) are doing this research study to:

- Learn more about pain management for teens undergoing spinal fusion surgery.
- Learn more about tools to help teens cope with stress and pain around surgery.
- Evaluate the effectiveness of a program to reduce acute and chronic pain and opioid use after surgery.
- Evaluate the effectiveness of a program to improve health-related quality of life after surgery.

How long would I be in the study and what will I need to do?

If you choose to take part in all the study assessments, you would be in the study for 7-11 months. All participants will be asked to complete online questionnaires about pain, mood, sleep, substance use, and health. This will be done 4 times during the study (before surgery, after hospital discharge, 3 months after surgery, and 6 months after surgery) and should take about 20-30 minutes to complete each time. At three of the four time points, teen participants

Seattle Children's IRB Approved
Effective: 4/28/2021

will be asked to complete brief (about 2 minutes) daily diary surveys for 1-2 weeks. In addition, we will check in with teen participants briefly via text message (<30 seconds each day) to see if they have been discharged.

All participants will also use an online program or mobile application for about 30 minutes each week for a month prior to the teen's surgery and for a month at home after the teen's surgery. There are two versions of the program. One program focuses on skills training to prepare for and recover after surgery. The other one focuses on education about pediatric spine surgery. The program you use will be selected by chance, like flipping a coin. Neither you nor the study doctor will choose what program you will use. You will have an equal chance of being given each treatment. The study team will check if you have logged in to the study program and which sections you have completed. All participants will complete a brief 5 minute survey after each section of the online program or mobile application to provide feedback on their experience.

Participating in this research study will not affect the medical care you receive at your hospital or your relationship with your doctor. Information collected from this study may be gathered and de-identified for use in future research without your additional informed consent.

What are the potential risks or discomforts if I join this study?

There are potential harms or risks if you take part in this study. Some of the questions you and your parent would be asked may seem personal, or make you uncomfortable. You may refuse to answer any questions that you do not want to answer.

There is a risk for loss of confidentiality or privacy. This would mean that someone other than the research team or our collaborators may find out that you were in the research or see your answers or medical information. We will make every effort to make sure that this does not happen.

A Data Safety Monitoring Board will review the information from this research study. This board is made of a group of experts. They are responsible for looking at how people in the research study are doing. If you take part, we would tell you about any new information we learn that might affect your health or your willingness to stay in the study.

Will being in this study benefit me in any way?

We cannot promise any benefits to you or others from your taking part in this research. Being in this study might benefit you in the following ways:

- Feeling more prepared for surgery.
- Feeling better after surgery.

Seattle Children's IRB Approved
Effective: 4/28/2021

We hope to use information we get from this study to benefit others undergoing major surgery. After the study is completed and results are published, we will send you an email about what we learned.

What are the alternatives if I choose not to participate?

Participation in research is completely voluntary. You can decide to participate or not to participate. Your alternative to participating in this research is not to participate.

Detailed Information: The following is more detailed information about this study in addition to the information listed above.

Why do I have the option of joining the study?

You have the option to take part in this research study because you (or your teen) will undergo spinal fusion surgery for idiopathic scoliosis, juvenile scoliosis, spondylolisthesis or kyphosis.

How many people will take part in the study?

We think that about 500 people will take part in this study from various hospitals and surgical centers around the country.

What are my responsibilities if I take part in this research?

If you take part in this research, you will be asked to:

- Complete study questionnaires and assessments privately (on your own) and to the best of your ability. You may still skip questions you do not want to answer.
- Contact study staff if you have any questions about what you have been asked to do.
- Be willing to comply with study procedures and communicate with study staff.
- Tell study staff if you are no longer interested in participating in the study.

What about confidentiality and privacy?

If you join the study, we will keep your information confidential as provided by law.

You have certain privacy rights regarding your Protected Health Information (PHI). Only with your permission may we create, use, or share your PHI for this study. The following describes the types of PHI the study will create, use, or share, who may use it or share it, and the purposes for which it may be used or shared.

PHI may include things like:

- Past or future medical records,

Seattle Children's IRB Approved
Effective: 4/28/2021

- Research records, such as surveys, questionnaires, interviews, or self-reports about medical history
- Medical or laboratory records related to this study, or
- Information specific to you like your name, address, birthday, ethnic origin, or identifying numbers like your social security number.

PHI may be created by, used by, or shared with:

- Researchers (such as doctors and their staff) taking part in this study here and at other centers,
- Research sponsors – this includes any persons or companies working for, with, or owned by the sponsor,
- Other people or organizations involved with your health care
- Review boards (such as Seattle Children's Institutional Review Board), data and safety monitoring boards, and others responsible for overseeing the conduct of research (such as monitors),
- Governmental agencies like the U.S. Food and Drug Administration (FDA), the Department of Health and Human Services (DHHS) and similar agencies in other countries, or
- Public health authorities to whom we are required by law to report information for the prevention or control of disease, injury, or disability.

PHI may be created, used, or shared to:

- Study the results of this research,
- Check if this study was done correctly,
- Complete and publish the results of the study described in this form,
- Comply with non-research obligations (such as notifying others if we think you or someone else could be harmed), or
- Facilitate your health care.

You may look at or copy the information that may be used or disclosed. However, for certain types of research studies, some of your PHI may not be available to you during the study. This does not affect your right to see what is in your medical (hospital) records.

Your permission for the use or sharing of your information will not expire, but you may cancel it at any time. You can do this by notifying the study team in writing. If you cancel your permission, no new information will be collected about you. However, information that has already been collected may still be used and shared with others.

Researchers continue to analyze data for many years, and it is not always possible to know when they will be done. If your information will be banked as part of this study, it may be used in the future for other research. We will not ask for your permission prior to this future research.

We will follow privacy laws when creating, using, or sharing your information, but these laws only apply to doctors, hospitals, and other health care providers. Some people who receive your health

Seattle Children's IRB Approved
Effective: 4/28/2021

information as part of this study may share it with others without your permission if doing so is permitted by the laws they must follow.

If the results of the study are published, information that identifies you will not be used.

Your permission is documented by signing this form below. If you decide that we cannot use or share your information, you cannot participate in this study.

NIH HEAL Initiative Data Storage and Sharing: Your de-identified study data will be stored securely at the University of Utah or at sites NIH selects for this study. Your de-identified data will be stored indefinitely. We will do our best to protect your personal information. Your name and other personally-identifying information will not be kept with the data. Your de-identified data will be stored with a code linking them to your identifying information. The key to the code will be kept at Seattle Children's Research Institute in a separate, secure area and will not be shared outside of Seattle Children's Research Institute.

This study is part of the NIH HEAL Initiative focused on understanding and developing new treatments for addiction and pain. Research gives us the best information and progresses more quickly when data is available from many studies and many individuals, and when many researchers can work with the data and analyze them in different ways. Therefore, your de-identified data will be used for this and other NIH HEAL Initiative studies. Your stored de-identified data will also be made widely available to other researchers. The shared de-identified data may be used indefinitely for research not related to this study or the HEAL Initiative, without asking you for additional consent.

If you withdraw from this research study before it is done, we may keep and continue to use de-identified data that have already been collected.

Potential benefits of sharing of data
There is no direct benefit to you from the storage and sharing of your de-identified data, but sharing may help researchers learn more about pain, health, and recovery after surgery, which may help you or others in the future.

Risks of sharing data
Even though we will protect your privacy as much as possible, there is a very small chance that the de-identified data could be identified as yours. The risk of this happening is very small, but may increase in the future as technology changes.

Research using data from this study may lead to new tests, drugs, or devices with commercial value. You will not receive any payment for any product developed from research using your data.

Seattle Children's IRB Approved
Effective: 4/28/2021

If you do not want your de-identified data used for other research, you should not participate in this study.

The creation, use or sharing of specific kinds of information requires that certain minors provide separate permission. Adolescents who are within the age ranges below will complete this section. For all other minor participants, the parent/legally authorized representative providing permission will complete this section on behalf of the child. Mark your permission with your initials below if you agree:

______*Behavioral or mental health/illness (age 13 and older)*
Initials
____ *Drug or alcohol abuse, diagnosis, or treatment (age 13 and older)*
Initials

Certificate of Confidentiality

We have a Certificate of Confidentiality from the federal government. It means we can't be forced to give out information about you if you take part in this study. This is true even if we are asked to by a court of law. It's not likely that someone would ask us to give out your personal information but this Certificate helps protect it. However, there are times when we would still need to share information about you.

Even with the Certificate, your information could still be given out under these situations:

- Federal agencies, like the FDA, may review study records
- Seattle Children's or the funding agency may look at study records to make sure the study is being done well
- You or a family member could share information about you or your part in this research study
- You give written permission to an insurer, employer or other person to receive information about you
- We must report child abuse or if you intend to hurt yourself or others

Would it cost me money to be in the study?

Seattle Children's IRB Approved
Effective: 4/28/2021

If you take part in this study, there would be no cost to you and no cost to you or your insurance company for the research procedures. Paying for your surgery and your follow-up care are your responsibility and are not part of the study.

What if I were injured because I joined the study?

If you think you have been harmed from this study, please call Dr. Jennifer Rabbitts at 206-884-1361 or email her at Jennifer.Rabbitts@seattlechildrens.org.

Would I be paid if I join this study?

To thank you for taking part in the study, we would give teen participants a total of $340 and parent/caregiver participants a total of $240 if all study assessments are completed. Payment would be given in the form of Amazon eGift cards. The payment is broken down as follows:

- **Assessment 1**: Each teen and parent will receive a $50 gift card after completion of their assessment.
- **Assessment 2**: Teens will receive a $50 gift card for **each** completed week of the 2-week daily assessments, for a total up to $100. Parents will receive $50 gift card for completing their survey assessment. Each teen and parent will receive $20 for completion of the pre-surgery website/app feedback survey.
- **Assessment 3**: Each teen and parent will receive a $50 gift card after completion of their assessment. Each teen and parent will receive $20 for completion of the post-surgery website/app feedback survey.
- **Assessment 4**: The teen will receive a $100 gift card, and the parent will receive a $50 gift card for completion of their final assessment.

The IRS has certain rules about paying people who take part in research studies. If you took part in this study, we would ask you to provide your name so we could pay you.

You can be in this study even if you do not give us this information. If you decide not to give us this information, you would receive no payment.

The payments you would receive for being in this study might be taxable. Seattle Children's is required to report to the IRS study payments totaling $600 or more made to anyone in any year.

If I join the study, can I stop, or can I be removed?

If you join the study, you can decide to stop **at any time for any reason**. If you decided to stop, you would need to talk with Dr. Jennifer Rabbitts so you leave the study in a safe way. You can contact this person by phone 206-884-1361 or jennifer.rabbitts@seattlechildrens.org

Seattle Children's IRB Approved
Effective: 4/28/2021

If you choose to leave the study, it will not affect your care at your hospital. You will not lose any benefits or be penalized if you choose to leave the study. You could be removed from the study if you do not complete the first set of surveys, develop a new medical condition, have a second major surgery during the study, or if your surgery is cancelled.

You could also be removed from the study by the research team if we decide that you didn't understand or comply with the study instructions.

Who do I contact if I have problems, questions or want more information?

This study has been reviewed and approved by an Institutional Review Board (IRB). You may contact the IRB, see the information below.

If I have questions or would like to know about …	You can call …	At …
• Emergencies<br>• General study questions<br>• Research-related injuries<br>• Any research concerns or complaints	**Jennifer Rabbitts**	**Phone: 206-884-1361**
• Emergencies<br>• General study questions<br>• Research-related injuries<br>• Any research concerns or complaints	**Tricia Fiddick**	**Phone: 206-884-1308**
• Your rights as a research participant<br>• Study questions, concerns or complaints.<br>• Contacting someone outside of study team	**Institutional Review Board This is a group of scientists and community members who make sure research meet legal and ethical standards.**	**Phone: (206) 987-7804**

Seattle Children's IRB Approved
Effective: 4/28/2021

More Information:
A description of this clinical trial will be available on http://www.ClinicalTrials.gov, as required by U.S. Law. This website will not include information that can identify you. At most, the website will include a summary of the results. You can search this website at any time.

What would my electronic signature on this form mean?

Your electronic signature on this form would mean:

- The research study was explained to you.
- You had a chance to ask all the questions you have at this time. All your questions have been answered in a way that is clear.
- You understand that the persons listed on this form will answer any other questions you may have about the study or your rights as a research study participant.
- **You have rights as a research participant. We will tell you about new information or changes to the study that may affect your health or your willingness to stay in the study.**
- By electronically signing this consent form, you do not give up any of your legal rights. The researcher(s) or sponsor(s) are not relieved of any liability they may have.
    - You agree to take part in the research study.
    - If the person reading this form is a parent/ legally authorized representative, you agree to have your teen take part in this research study.
    - You permit the creation, use, and sharing of your health information for the purposes of this research study as described in the "**What about confidentiality and privacy?" and "NIH HEAL Initiative Data Storage and Sharing"** section above.

Please Note: If the person taking part in this research study is a foster child or a ward of the state, then please tell the researcher or their staff.

Seattle Children's IRB Approved
Effective: 4/28/2021
